# Exploring the Role of Bifenthrin in Recurrent Implantation Failure and Pregnancy Loss Through Network Toxicology and Molecular Docking

**DOI:** 10.3390/toxics13060454

**Published:** 2025-05-29

**Authors:** Shengyuan Jiang, Yixiao Wang, Haiyan Chen, Yuanyuan Teng, Qiaoying Zhu, Kaipeng Xie

**Affiliations:** 1 Department of Obstetrics and Gynecology, Women’s Hospital of Nanjing Medical University, Nanjing Women and Children’s Healthcare Hospital, Nanjing 210004, China; jiangsy0322@163.com (S.J.); 13770982878@163.com (Y.W.); haiyan.chen2022@stu.njmu.edu.cn (H.C.); 2 Department of Public Health, Women’s Hospital of Nanjing Medical University, Nanjing Women and Chidren’s Healthcare Hospital, Nanjing 210004, China; tyy130023@163.com

**Keywords:** bifenthrin, recurrent implantation failure, recurrent pregnancy loss, endocrine-disrupting chemical, molecular docking

## Abstract

Bifenthrin (BF) is a widely used pyrethroid pesticide recognized as an endocrine-disrupting chemical (EDC). Previous studies have confirmed that chronic exposure to BF is associated with various health risks. However, its potential association with recurrent implantation failure (RIF) and recurrent pregnancy loss (RPL) remains unclear. In this study, the potential targets of BF were identified using several databases, including the Comparative Toxicogenomics Database (CTD), TargetNet, GeneCards, SwissTargetPrediction, and STITCH. Differentially expressed genes (DEGs) associated with RIF were obtained from bulk RNA-seq datasets in the GEO database. Candidate targets were identified by intersecting the predicted BF-related targets with the RIF-associated DEGs, followed by functional enrichment analysis using the DAVID and g:Profiler platforms. Subsequently, hub genes were identified based on the STRING database and Cytoscape. A diagnostic model was then constructed based on these hub genes in the RIF cohort and validated in an independent recurrent pregnancy loss (RPL) cohort. Additionally, we performed single-cell type distribution analysis and immune infiltration profiling based on single-cell RNA-seq and bulk RNA-seq data, respectively. Molecular docking analysis using AutoDock Vina was conducted to evaluate the binding affinity between BF and the four hub proteins, as well as several hormone-related receptors. Functional enrichment results indicated that the candidate genes were mainly involved in apoptotic and oxidative stress-related pathways. Ultimately, four hub genes—BCL2, HMOX1, CYCS, and PTGS2—were identified. The diagnostic model based on these genes exhibited good predictive performance in the RIF cohort and was successfully validated in the RPL cohort. Single-cell transcriptomic analysis revealed a significant increase in the proportion of myeloid cells in RPL patients, while immune infiltration analysis showed a consistent downregulation of M2 macrophages in both RIF and RPL. Moreover, molecular docking analysis revealed that BF exhibited high binding affinity to all four hub proteins and demonstrated strong binding potential with multiple hormone receptors, particularly pregnane X receptor (PXR), estrogen receptor α (ESRα), and thyroid hormone receptors (TR). In conclusion, the association of BF with four hub genes and multiple hormone receptors suggests a potential link to immune and endocrine dysregulation observed in RIF and RPL. However, in vivo and in vitro experimental evidence is currently lacking, and further studies are needed to elucidate the mechanisms by which BF may contribute to RIF and RPL.

## 1. Introduction

Adverse reproductive outcomes, including recurrent implantation failure (RIF) and recurrent pregnancy loss (RPL), continue to pose significant challenges in the field of reproductive medicine. RIF is characterized by the inability to achieve pregnancy following the transfer of one or two high-quality embryos in three or more consecutive assisted reproductive cycles, such as in vitro fertilization (IVF) or intracytoplasmic sperm injection (ICSI) [1]. This condition may be attributed to factors such as endometrial dysfunction, ovarian insufficiency, or suboptimal sperm quality. RPL is defined as the occurrence of two or more consecutive miscarriages before 28 weeks of gestation with the same partner [2] and is frequently associated with chromosomal abnormalities, uterine or placental anomalies, endocrine disorders, or infections. Both RIF and RPL have been linked to immune dysregulation and imbalances in the vaginal–uterine microbiota [3,4].

Endocrine-disrupting chemicals (EDCs) constitute a group of synthetic or naturally occurring chemicals capable of interfering with hormonal levels in organisms, thereby disrupting endocrine system functions and negatively affecting reproductive, developmental, neurological, and immunological health [5]. Mounting evidence suggests that long-term exposure to EDCs, such as bisphenol A (BPA), may lead to neurotoxic effects [6]. Notably, several EDCs have been implicated in adverse neurodevelopmental outcomes in children [7]. Furthermore, exposure to di-(2-ethylhexyl) phthalate (DEHP) has been potentially associated with neurocognitive impairments in middle-aged and elderly populations [8]. Additionally, EDCs exhibit significant immunotoxic effects. Compounds such as bi-sphenols (BPA, BPAF, and BPS), estrogen-active chemicals (17β-estradiol, diethylstil-bestrol, and zearalenone), some pesticides (fungicide vinclozolin, herbicide atrazine, and insecticide cypermethrin), and other environmental pollutants (17α-ethinylestradiol, diethyl phthalate, and perfluorooctane sulfonate) have all been demonstrated to induce immunological dysfunction by modulating the receptor for activated C kinase 1 (*RACK1*) expression [9,10,11,12]. Additionally, pyrethroid insecticides, such as cypermethrin, deltamethrin, and permethrin, have also been found to exhibit immunotoxic effects [13,14,15]. Concerning the reproductive system, numerous studies have confirmed that EDCs such as perfluorinated compounds, BPA, and phthalates interfere with ovarian function and gonadal development [16,17].

Bifenthrin (BF), a common pyrethroid-class EDC belonging to the type I pyrethroids, is extensively used in agriculture and vector control [18]. Due to its low water solubility, low volatility, and strong adsorption properties in soil and sediments, BF exhibits significant environmental persistence and bioaccumulative potential [19,20]. Human exposure primarily occurs via ingestion, inhalation, and dermal contact [21]. Environmental monitoring data indicate that BF concentrations have exceeded surface water safety thresholds in agricultural waters across multiple European countries, including Spain, France, and Switzerland, as well as in Argentina [22]. A study conducted in Southeastern China reported average residual concentrations of BF in surface soils and surface waters of 12.14 ng/g and 3.36 µg/L, respectively [23]. Similarly, research in India evaluating pesticide residues in breast milk revealed higher levels of BF exposure among urban residents compared to rural populations [24]. These findings collectively suggest that BF contamination has emerged as a global environmental concern.

As an EDC, BF may exert distinct effects across different species or even at different developmental stages within the same species. Previous studies have demonstrated its estrogenic and anti-estrogenic activities, capable of disrupting the hypothalamic–pituitary–gonadal (HPG) axis in fish and mammals, thereby influencing endocrine system functions [25,26]. Besides estrogenic effects, BF can also disturb the hypothalamic–pituitary–thyroid (HPT) axis, impairing thyroid function [27]. Additionally, BF may inhibit cortisol and aldosterone synthesis by disrupting the cAMP signaling pathway [28]. Beyond endocrine disruption, BF affects organismal function through other mechanisms. For instance, it induces oxidative stress and immunotoxicity, as evidenced by changes in catalase (CAT) and glutathione peroxidase (GPX) activity in mouse livers [29]. Studies have also reported BF-induced immunotoxicity and increased mortality in fish [30]. In embryonic development, BF can cause embryotoxicity in zebrafish, triggering inflammatory cell death and antiangiogenic effects [31].

Previous studies have demonstrated that BF can adversely affect reproductive function in various organisms. For instance, research by Wonhyoung et al. revealed that BF inhibited the proliferation of porcine trophoblast (pTr) and uterine luminal epithelial (pLE) cells, thereby diminishing the potential for embryo implantation [32]. Mechanistically, these effects are believed to involve mitochondrial dysfunction, reactive oxygen species (ROS) generation, endoplasmic reticulum stress, calcium imbalance, and *MAPK/PI3K* signaling pathway disruption [32]. Such pathological changes—particularly inflammatory responses, oxidative stress, and apoptosis in endometrial stromal and trophoblast cells—are closely linked to recurrent RIF and RPL [4,33,34].

Currently, no studies have yet investigated the association between BF and adverse reproductive outcomes in humans. This study aims to further explore potential associations between BF exposure and human reproductive health through bioinformatics and molecular docking methodologies.

## 2. Methods

**Figure 1:**
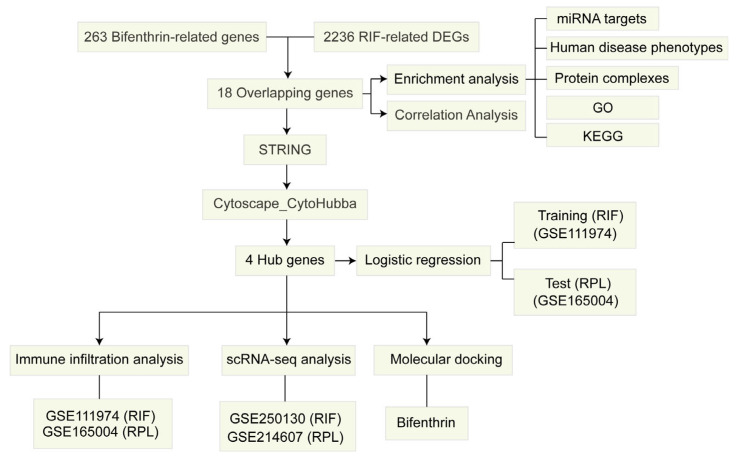
Flowchart of the study design.

The flowchart of the present bioinformatic-based toxicology investigation is shown in Figure 1.

### 2.1. Identification of BF-Associated Genes

To identify potential target genes associated with BF, we collected data from five comprehensive databases: the Comparative Toxicogenomics Database (CTD), TargetNet, GeneCards, SwissTargetPrediction, and STITCH. After compiling the initial gene lists from these sources, we performed data merging and removed duplicate entries to create a consolidated set of unique target genes.

### 2.2. Acquisition of RIF and RPL RNA-seq Data

All datasets utilized in this study, including both bulk RNA-seq and single-cell RNA-seq (scRNA-seq) data, were obtained from the Gene Expression Omnibus (GEO) database. We developed a logistic regression diagnostic model using the GSE111974 dataset, which was subsequently validated in the independent GSE165004 cohort. Detailed information about the datasets is presented in Table 1.

### 2.3. Approach to Identifying Hub Genes 

Differential expression analysis was conducted on the GSE111974 dataset using the following thresholds: |log_2_FoldChange| > 0.59 (corresponding to |fold change| > 1.5) and adjusted *p*-value < 0.05. The resulting differentially expressed genes (DEGs) were cross-referenced with BF-associated genes to identify overlapping candidates. These overlapping genes were then uploaded to the STRING database to construct a protein–protein interaction (PPI) network. Subsequently, hub genes were identified using eight metrics provided by the CytoHubba plugin: Stress, Radiality, Maximum Neighborhood Component (MNC), Maximal Clique Centrality (MCC), Edge Percolated Component (EPC), Degree, Closeness, and Betweenness.

### 2.4. Functional Enrichment Analysis

Enrichment analyses of miRNA targets, transcription factor binding motifs, tissue-specific expression, protein complexes, and human disease phenotypes were performed using g:Profiler (https://biit.cs.ut.ee/gprofiler/gost (accessed on 10 May 2025)). The overlapping genes were analyzed for Gene Ontology (GO) terms and Kyoto Encyclopedia of Genes and Genomes (KEGG) pathways using the DAVID database. Results were visualized using the ggplot2 package in R.

### 2.5. Construction and Evaluation of the Predictive Model

A logistic regression model was constructed in the RIF cohort based on four selected hub genes using the glm function in R, and a nomogram was generated to estimate RIF risk. Model performance was assessed through calibration curves, decision curve analysis (DCA), and receiver operating characteristic (ROC) curves. To further validate model robustness, we applied the model to the RPL cohort. Additionally, using the glm and pROC packages in R, we generated 1000 random 4-gene combinations to construct logistic regression models and calculate AUC values, with model performance evaluated in both training and test sets.

### 2.6. scRNA-seq Analysis of RIF and RPL

We obtained datasets from the GEO database. The RIF cohort was derived from GSE250130, comprising over 220,000 single cells from the endometrium of six healthy women and ten patients with RIF. The RPL cohort was obtained from GSE214607, including over 110,000 single cells from the decidua of five women with normal pregnancies and three patients with RPL.

Quality control of single-cell RNA-seq data involved removing cells with <400 or >6000 detected genes or >20% mitochondrial gene content. Data normalization and variance stabilization were performed using the NormalizeData function (v2 algorithm, Seurat v4). Batch effects were corrected using the Harmony algorithm. The optimal number of principal components was selected based on the ElbowPlot (cumulative variance > 90%, individual variance <5%). Cell clustering was performed using FindNeighbors and FindClusters (Louvain algorithm), and UMAP was used for dimensionality reduction and visualization. Marker genes were identified via FindAllMarkers, and we identified lineage markers using CellMarker2 and annotated cell types accordingly. Gene set activity was assessed with AddModuleScore, and average gene expression per cluster and condition was calculated using AverageExpression. Detailed information about the datasets is presented in Table 1.

### 2.7. Immune Infiltration and Correlation Analysis

We employed the CIBERSORT algorithm, an in silico computational approach, to quantify the relative proportions of 22 immune cell subtypes in both RIF and RPL samples. Low-abundance immune cell subtypes were excluded. Immune infiltration patterns were compared between patient groups (RIF/RPL) and healthy controls using Wilcoxon rank sum tests. Furthermore, we investigated potential relationships between the four hub genes and immune cell infiltration profiles through Spearman correlation analysis.

### 2.8. Molecular Docking

BF’s 3D structure was generated from PubChem (https://pubchem.ncbi.nlm.nih.gov/ (accessed on 1 March 2025)). Protein targets were retrieved from RCSB PDB and prepared in PyMOL by removing solvent molecules and non-essential groups. Structural preparation for docking included hydrogen addition and ligand torsion optimization using AutoDock tools. Protein–ligand interactions were analyzed using AutoDock Vina 1.5.6 [35], with binding conformations evaluated by docking scores. Molecular interactions were visualized and analyzed through PyMOL and Discovery Studio 2019, producing comprehensive 2D and 3D interaction models.

### 2.9. Statistical Analysis

All statistical analyses and visualizations were performed in R software (version 4.3.3). Differential expression was analyzed using the “limma” package. The Wilcoxon rank sum test was applied to compare immune cell infiltration between groups. Data visualization was conducted using “ggplot2”. A *p*-value > 0.05 was considered not statistically significant.

## 3. Results

### 3.1. Identification of Hub Genes and Enrichment Analysis

**Figure 2:**
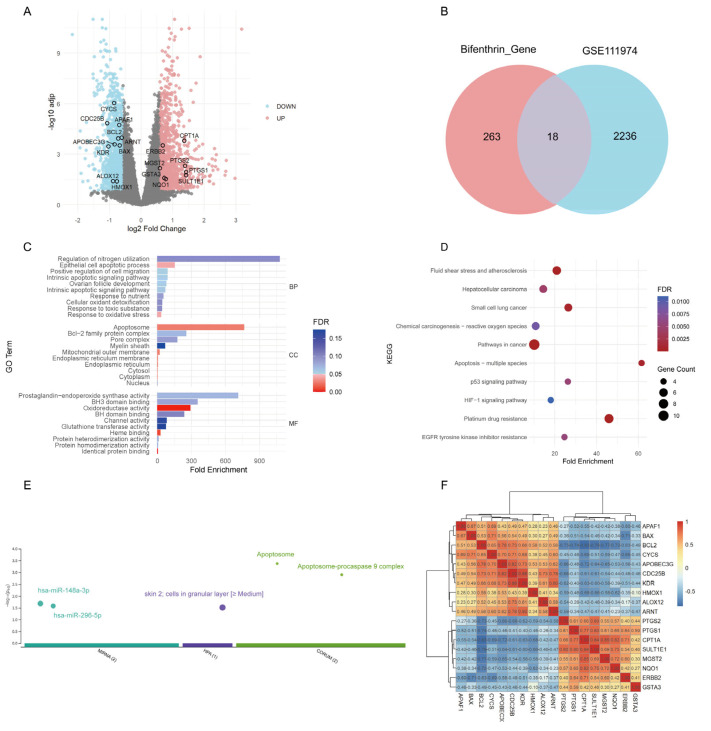
Analysis of bifenthrin-associated genes and DEGs in the RIF cohort. (**A**) Volcano plot showing the DEGs in the RIF cohort (GSE111974) identified by “limma” analysis. (**B**) Venn diagram showing the overlap between bifenthrin-targeted genes and RIF-associated DEGs. (**C**) g:Profiler enrichment analysis of the 18 overlapping genes. (**D**) GO enrichment analysis of the 18 overlapping genes. (**E**) KEGG pathway enrichment analysis of the 18 overlapping genes. (**F**) Spearman correlation heatmap of the 18 target genes. Abbreviations: DEG, differentially expressed gene; RIF, recurrent implantation failure; GO, Gene Ontology; KEGG, Kyoto Encyclopedia of Genes and Genomes.

Integrated analysis of five databases identified 263 BF-related targets (Supplementary Table S1). Bulk RNA-seq analysis of RIF samples (GSE111974) identified 2236 differentially expressed genes (DEGs), including 1013 upregulated and 1223 downregulated genes (Figure 2A). By intersecting the DEGs from the RIF dataset with BF-associated genes, we identified 18 overlapping genes (Figure 2B). GO enrichment analysis revealed that these 18 genes were primarily involved in apoptotic processes and oxidoreductase activity (Figure 2C), while KEGG pathway analysis indicated enrichment in pathways such as fluid shear stress and atherosclerosis, as well as apoptosis (Figure 2D). The gProfiler enrichment analysis revealed that the 18 target genes were significantly enriched in multiple biological databases. miRNA targets analysis suggests that these genes may be regulated by hsa-miR-148a-3p and hsa-miR-296-5p. Human disease phenotypes analysis indicate that these genes are highly expressed in granular layer cells of the epidermis. Protein complexes analysis further shows that they are involved in the formation of the apoptosome and its complex with procaspase 9, suggesting a potential role in apoptosis (Figure 2E). The correlations among the 18 genes are shown in Figure 2F. Among these genes, *CYCS*, *BCL2*, and *HMOX1* exhibited significant positive correlations with each other, whereas *PTGS2* showed a negative correlation with the other three genes.

### 3.2. Identification of Hub Genes

**Figure 3:**
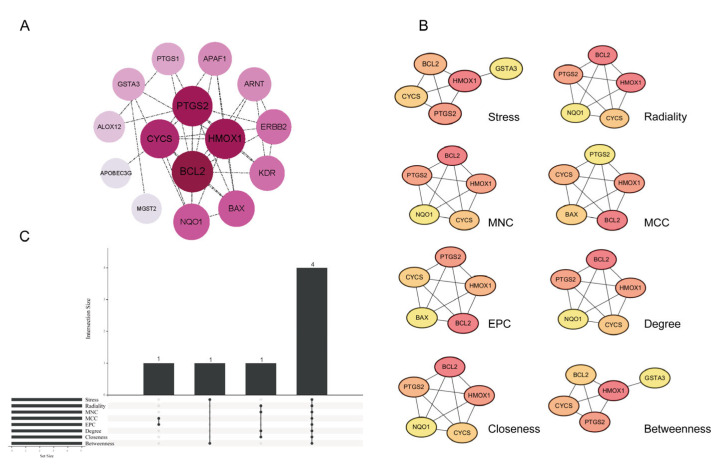
Identification of hub genes. (**A**) PPI network ranked by Degree value. (**B**) Genes screened through eight network metrics. (**C**) UpSet plot displaying overlapping genes identified by eight network metrics in CytoHubba. Abbreviations: MNC, Maximum Neighborhood Component; MCC, Maximal Clique Centrality; EPC, Edge Percolated Component.

The 18 candidate genes were analyzed using the STRING database to construct a protein–protein interaction (PPI) network, which comprised 15 nodes and 38 edges (Figure 3A). We applied eight different network metrics to rank the importance of 18 candidate genes, including Stress, Radiality, MNC, MCC, EPC, Degree, Closeness, and Betweenness. The top five genes identified by each metric were selected (Figure 3B), and an intersection analysis was performed (Figure 3C). As a result, four common hub genes were identified: *PTGS2*, *CYCS*, *BCL2*, and *HMOX1*.

### 3.3. Construction and Evaluation of the Four-Gene Model

**Figure 4:**
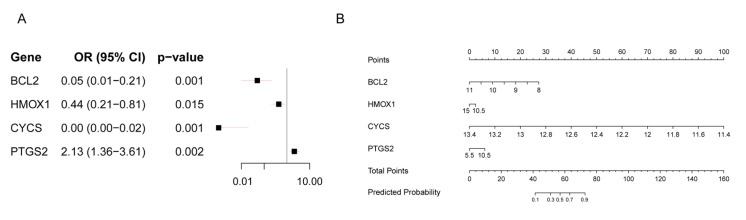
Logistic Regression Prediction Model of the RIF cohort. (**A**) Forest plot of univariate logistic regression analysis for four hub genes in the RIF cohort. (**B**) Nomogram of the prediction model.

Univariate logistic regression analysis revealed that lower expression levels of *BCL2* (OR: 0.05, 95% CI: 0.01–0.21; *p* < 0.001), *HMOX1* (OR: 0.44, 95% CI: 0.21–0.81; *p* = 0.015), and *CYCS* (OR: 0.00, 95% CI: 0.00–0.02; *p* < 0.001) were significantly associated with a reduced risk of RIF, while higher expression of PTGS2 (OR: 2.13, 95% CI: 1.36–3.61; *p* = 0.002) was positively associated with RIF risk (Figure 4A). Based on these four hub genes (*BCL2*, *HMOX1*, *CYCS*, and *PTGS2*), we developed a predictive model for the RIF cohort. A nomogram was constructed to visualize the model and aid in clinical risk assessment (Figure 4B).

**Figure 5:**
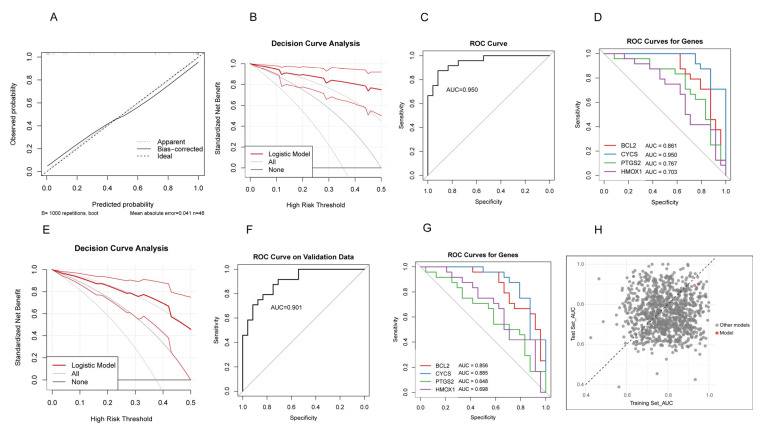
Evaluation of the predictive model. (**A**) Calibration curve in the RIF cohort. (**B**) Decision curve analysis (DCA) in the RIF cohort. (**C**) ROC curve in the RIF cohort. (**D**) ROC curves of the four hub genes in the RIF cohort. (**E**) DCA curve in the RPL cohort. (**F**) ROC curve in the RPL cohort. (**G**) ROC curves of the four hub genes in the RPL cohort. (**H**) Scatter plot of AUC values from 1000 models constructed by randomly selecting four genes in the RIF training set and applying to the RPL test set. The red dot represents the model based on PTGS2, BCL2, HMOX1, and CYCS.

The calibration curve in the RIF cohort demonstrated excellent agreement between predicted probabilities and observed outcomes, indicating good model calibration (Figure 5A). DCA showed significant clinical net benefit across a wide range of threshold probabilities (Figure 5B). ROC analysis further confirmed the model’s strong discriminative performance, with an AUC of 0.950 (Figure 5C). The individual predictive performance of each gene was as follows: *BCL2* (AUC = 0.861), *HMOX1* (AUC = 0.703), *CYCS* (AUC = 0.950), and *PTGS2* (AUC = 0.767) (Figure 5D).

The RPL cohort demonstrated that the predictive model maintained robust discriminative performance. The DCA confirmed the model’s reliability (Figure 5E). ROC analysis showed an AUC of 0.901 for the overall model (Figure 5F). The individual diagnostic performance of each gene was as follows: *BCL2* (AUC = 0.856), *HMOX1* (AUC = 0.698), *CYCS* (AUC = 0.885), and *PTGS2* (AUC = 0.648) (Figure 5G). We randomly selected four genes 1000 times from the RIF cohort to construct predictive models and applied these models to the RPL cohort, calculating the AUC values for each iteration. The model based on *PTGS2*, *BCL2*, *HMOX1*, and *CYCS* consistently demonstrated high AUCs in both the RIF and RPL cohorts, indicating strong stability and predictive performance (Figure 5H). These results indicate that the model also exhibits strong diagnostic potential in the RPL population, suggesting that despite clinical differences between RIF and RPL, this gene panel may hold shared predictive value across both conditions.

### 3.4. Single-Cell RNA-seq Analysis of RIF and RPL

**Figure 6:**
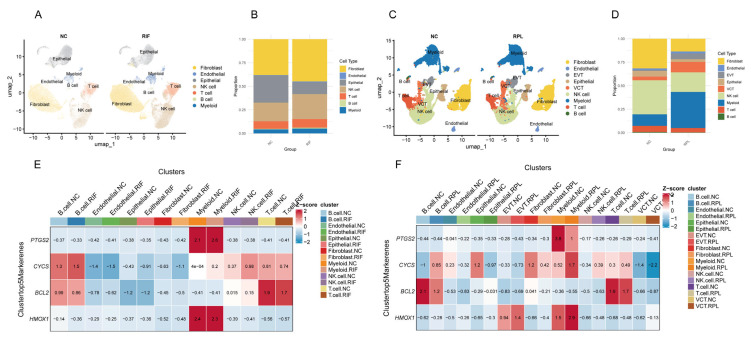
scRNA-seq analysis of RIF and RPL. (**A**) UMAP visualization of cellular heterogeneity in the RIF cohort. (**B**) Compositional bar plot depicting cell type proportions in the RIF cohort. (**C**) UMAP visualization of cellular heterogeneity in the RPL cohort. (**D**) Compositional bar plot depicting cell type proportions in the RPL cohort. (**E**) Z-score heatmap showing the expression patterns of four hub genes across different cell types in the RIF cohort. (**F**) Z-score heatmap showing the expression patterns of four hub genes across different cell types in the RPL cohort.

We performed batch effect correction on the RIF and RPL datasets (Supplementary Figure S1), followed by cell clustering analysis. The results revealed that in the RIF cohort, fibroblasts, epithelial cells, and NK cells accounted for a larger proportion (Figure 6A,B), whereas in the RPL cohort, the predominant cell populations were fibroblasts, NK cells, and myeloid cells (Figure 6C,D). Notably, there were no statistically significant differences in the proportions of cell types in the RIF cohort. However, the proportion of myeloid cells was significantly increased in the RPL cohort compared to the control group (*p* < 0.05), while differences in other cell types were not statistically significant (Supplementary Figure S2).

To visualize expression trends, we generated a Z-score heatmap illustrating the relative expression patterns of four key genes across different cell types. *PTGS2* showed consistently high expression in myeloid cells in both cohorts. *CYCS* was predominantly expressed in T cells, while *BCL2* exhibited elevated expression in both B and T cells. *HMOX1* was mainly expressed in myeloid cells, with notably increased expression in EVT cells within the RPL cohort (Figure 6E,F).

### 3.5. Immune Cell Infiltration Analysis

**Figure 7:**
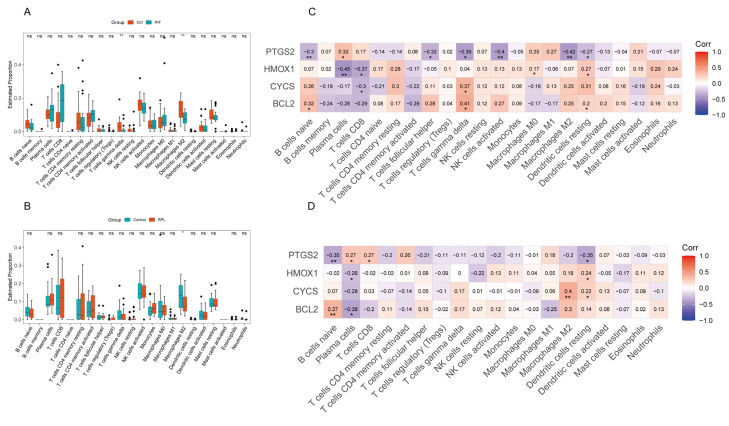
Analysis of immune cell infiltration in the RIF and RPL cohort. (**A**) Boxplots of immune infiltration analysis for immune cells in the RIF cohort. (**B**) Boxplots of immune infiltration analysis for immune cells in the RPL cohort. (**C**) Heatmap of the Spearman correlation between PTGS2, HMOX1, CYCS, BCL2, and immune cells in the RIF cohort. (**D**) Heatmap of the Spearman correlation between PTGS2, HMOX1, CYCS, BCL2, and immune cells in the RPL cohort. * *p* < 0.05, ** *p* < 0.01.

To further explore the relationship between adverse reproductive outcomes and four genes with 22 immune cell subtypes, we used the CIBERSORT algorithm to analyze the infiltration of 22 immune cell types in RIF and RPL. Compared to controls, γδ T cells and M2 macrophages were significantly downregulated in patients with RIF (Figure 7A). In patients with RPL, M2 macrophages showed marked downregulation (Figure 7B).

We further analyzed the correlations between the four hub genes and immune cell infiltration and found that they exhibited several similar association patterns in both RIF and RPL. For example, *PTGS2* showed negative correlations with naïve B cells and resting dendritic cells but a positive correlation with plasma cells in both pathological states. *HMOX1* was negatively correlated with plasma cells and positively correlated with resting dendritic cells in both groups. Similarly, *BCL2* was positively correlated with naïve B cells in both cohorts. In contrast, *CYCS* did not display a consistent correlation pattern (Figure 7C,D).

In RIF, PTGS2 also showed negative correlations with follicular helper T cells, gamma delta T cells, activated NK cells, and M2 macrophages. *HMOX1* was negatively correlated with CD8+ T cells and positively correlated with M0 macrophages. *CYCS* was negatively correlated with CD8+ T cells but positively correlated with gamma delta T cells. *BCL2* showed positive correlations with both gamma delta T cells and resting dendritic cells. In RPL, *PTGS2* was positively correlated with plasma cells and CD8+ T cells, whereas *CYCS* was positively correlated with M2 macrophages and resting dendritic cells. In contrast, *BCL2* was negatively correlated with plasma cells (Figure 7C,D).

Overall, *PTGS2* exhibited an immune correlation pattern opposite to that of the other three hub genes in both RIF and RPL. Its expression levels consistently showed an opposite trend compared to the others in both pathological conditions. These findings suggest that the four hub genes may contribute to the pathogenesis of RIF and RPL by modulating distinct immune microenvironments.

### 3.6. Molecular Docking of BF with Four Proteins

To assess the binding affinity of BF with key molecular targets, molecular docking was conducted with four hub proteins (*PTGS2, HMOX1, CYCS*, and *BCL2*). A binding energy of <−7 kcal/mol was considered indicative of moderate affinity, −7 to −4 kcal/mol moderate, and >−4 kcal/mol weak or negligible binding. The results indicated that BF exhibited relatively strong binding affinity with all four targets: *PTGS2* (−8.7 kcal/mol), *HMOX1* (−8.3 kcal/mol), *BCL2* (−7.6 kcal/mol), and *CYCS* (−6.9 kcal/mol).

Given that BF may not directly bind to the hub proteins *PTGS2*, *HMOX1*, *CYCS*, and *BCL2*, and that existing studies have demonstrated that these hub genes are regulated by various hormone receptors, we hypothesized that BF may exert its effects through indirect mechanisms. For example, the progesterone receptor (PGR) regulates *PTGS2* expression [36]; estrogen-related receptor alpha (ERRα) and the aryl hydrocarbon receptor (AhR) regulate *HMOX1* [37,38]; the glucocorticoid receptor (GR) modulates *BCL2* [39]; and ERRα also regulates *CYCS* expression [40]. Therefore, we speculate that BF may indirectly influence the expression of these hub genes by interfering with the function of hormone receptors.

In addition, hormone receptors play crucial roles in female pregnancy-related physiological processes. Estrogen and progesterone are essential for endometrial decidualization and the maintenance of early pregnancy [41]; GR is involved in maternal–fetal immune tolerance; thyroid hormone receptors (TRs) maintain maternal–fetal metabolic homeostasis and influence fetal development [42,43]; the ERR family is critical for maintaining trophoblast stem cell function [44]; and the pregnane X receptor (PXR) regulates maternal vascular function and steroid metabolism during pregnancy [45,46].

As BF is a known EDC, this study aimed to investigate its potential reproductive toxicity by analyzing its binding affinity with various hormone receptors via molecular docking. A total of 12 receptors were selected as docking targets, including estrogen receptor alpha (ESRα), estrogen receptor beta (ESRβ), PGR, G protein-coupled estrogen receptor (GPER), GR, AhR, TRα, TRβ, ERRα, estrogen-related receptor beta (ERRβ), estrogen-related receptor gamma (ERRγ), and PXR (Table 2). To validate the docking results, we also performed molecular docking between each of the aforementioned receptors—namely ESRα, ESRβ, PGR, GPER, GR, AhR, TRα, and TRβ—and their respective specific endogenous ligands as positive controls.

The results showed that BF bound to ESRα with a binding energy of –8.1 kcal/mol, comparable to its natural ligand 17β-estradiol (E2, −9.9 kcal/mol). Similarly, BF bound to GPER with a binding energy of –7.6 kcal/mol, close to that of E2 (−7.9 kcal/mol), suggesting potential estrogenic or anti-estrogenic activity. Furthermore, BF showed strong binding to TRα (−8.8 kcal/mol), TRβ (−10.3 kcal/mol), and PXR (−10.6 kcal/mol). Moderate binding affinities were observed for ESRβ (–6.8 kcal/mol), PGR (−6.6 kcal/mol), and AhR (−6.5 kcal/mol). For the orphan nuclear receptors that lack well-defined endogenous ligands, BF exhibited strong binding to ERRα (−7.1 kcal/mol) and ERRγ (−7.8 kcal/mol), and moderate binding to ERRβ (−6.6 kcal/mol). The detailed amino acid interactions between BF and each receptor are provided in Supplementary Figure S3.

## 4. Discussion

RIF and RPL are two significant challenges in reproductive medicine, both characterized by an inability to achieve or sustain pregnancy. Although they differ in clinical presentation—RIF refers to multiple failed embryo transfers, while RPL involves consecutive natural miscarriages—they may share overlapping molecular mechanisms related to endometrial receptivity, immune microenvironment, and cellular stress responses.

This study integrates transcriptomic data from the endometrium of RIF and RPL patients and investigates potential mechanisms related to BF exposure, focusing on relevant target genes. Bio-enrichment analysis revealed that intersecting genes were significantly associated with oxidative stress and apoptosis pathways, highlighting their key role in embryo implantation.

RIF and RPL represent two significant challenges in reproductive medicine. Our enrichment analysis showed that the 18 BF-related genes were primarily associated with apoptotic processes and oxidative stress, which is consistent with previous studies [32]. However, due to the limited number of genes involved in the enrichment, we cannot definitively conclude that BF exerts its effects through modulation of apoptosis and oxidative stress in placental or uterine tissues. This hypothesis requires further validation through in vitro experiments.

We selected four hub genes from a set of 18 candidate genes. Our study found that *PTGS2* was upregulated in both RIF and RPL. As a rate-limiting enzyme in prostaglandin synthesis, *PTGS2* is critical in embryo implantation and decidualization. Its overexpression may trigger aberrant inflammatory responses, disrupt endometrial immune homeostasis and receptivity, and increase the risk of pregnancy failure [47]. Previous research has shown that *PTGS2* expression is elevated in mouse neural cells following 24-h exposure to BF, accompanied by increased oxidative stress [48]. In contrast, another study found that BF can inhibit luteinizing hormone-induced *PTGS2* expression via the protein kinase A (PKA) signaling pathway, resulting in ovulatory dysfunction [49]. In addition, we observed that *CYCS*, *HMOX1*, and *BCL2* were consistently downregulated in both RIF and RPL. *CYCS* encodes cytochrome c, a key factor in the mitochondrial electron transport chain and the intrinsic apoptotic pathway [50]. Contrary to our findings, BF has been reported to upregulate *CYCS* expression in mouse testicular cells, triggering mitochondrial-mediated apoptosis [51]. However, another pyrethroid—deltamethrin—was shown to significantly reduce *CYCS* expression in mouse placental tissue [52], suggesting that the regulation of *CYCS* may depend on the specific pyrethroid compound and target tissue involved. *HMOX1*, a metabolic enzyme with antioxidant, anti-apoptotic, and anti-inflammatory properties [53], is reportedly upregulated in macrophages upon BF exposure [54], while deltamethrin downregulates *HMOX1* in the placenta [52]. *BCL2*, a classic anti-apoptotic gene that maintains cell survival by inhibiting the mitochondrial apoptotic pathway [55], is also downregulated by BF in mouse hepatocytes [56] and by cypermethrin in human trophoblast cells, thereby promoting apoptosis [57]. These findings suggest that the pyrethroid regulation of *PTGS2, CYCS, HMOX1*, and *BCL2* is subject to substantial tissue specificity and interspecies variation, which may account for the inconsistent results across different studies.

Fangfang Li et al. demonstrated a global reduction in macrophage abundance in RIF through GSEA analysis [58]. Our study further used the CIBERSORT algorithm to dissect the distribution of macrophage subtypes. We found no significant differences in M0 or M1 macrophage levels but observed a marked reduction in M2 macrophages in both RIF and RPL. Previous studies have shown that M2 macrophage polarization plays a pivotal role in maintaining maternal–fetal immune tolerance [59]. These results indicate that impaired immune tolerance at the maternal–fetal interface may be a shared pathological mechanism in RIF and RPL. Also, regulating M2 polarization could represent a potential therapeutic target. Myeloid cells were significantly increased in the endometria of patients with RPL, according to single-cell transcriptomic data. However, immune infiltration analysis revealed decreased M2 macrophages, suggesting that other myeloid subsets may also contribute to pregnancy failure. For example, M-MDSCs (monocytic myeloid-derived suppressor cells) have been reported to be significantly elevated in both RIF and RPL [60]. Nonetheless, we did not detect substantial changes in other immune cell types, which may be due to the limited sample size in this study. This highlights the need for larger-scale investigations into the immunological basis of adverse pregnancy outcomes.

By integrating single-cell RNA sequencing and immune infiltration analysis, we found that *PTGS2*, *CYCS*, and *HMOX1* were highly expressed in endometrial immune cells, including B cells, T cells, and myeloid cells. Further correlation analysis revealed that *PTGS2* expression negatively correlates with M2 macrophage abundance in RIF, whereas *CYCS* expression shows a positive correlation with M2 macrophages in RPL, suggesting that these genes could serve as potential biomarkers and therapeutic targets for RIF and RPL, respectively.

Importantly, as an EDC, BF may induce immunotoxicity not only by modulating these hub genes but also by interfering with hormone signaling. Previous studies have shown that BF can activate estrogen receptors and regulate the expression of *RACK1*, thereby altering the embryonic immune microenvironment [5]. These results suggest that EDC exposure may disrupt pregnancy-related immune balance through multiple mechanisms that warrant further investigation.

We performed molecular docking between BF and four hub proteins (*PTGS2, CYCS, HMOX1*, and *BCL2*) and found high binding affinities. However, there is currently no direct evidence that BF exerts biological effects by directly binding to these proteins. Steroid hormone receptors play a central role in regulating endocrine homeostasis and inflammation [61], and are implicated in RIF pathogenesis. For example, loss of GR impairs decidualization and leads to implantation failure [62]. Furthermore, PGR deficiency enhances gonadotropin-induced *PTGS2* expression and indirectly suppresses *PTGS2* via the inhibition of NF-κB signaling [36]. *HMOX1* expression is regulated by receptors such as ERRα, AhR, and ESRα [37,38], while *BCL2* is modulated by ESRs [63]. The expression of GPER has also been shown to regulate the expression of *PTGS2* [64] and *HMOX1* [65]. In addition, given the hormone-like activity of BF, we performed molecular docking analyses between BF and 12 hormone receptors.

We found that BF exhibited strong binding affinity to PXR. Previous studies have reported that continuous exposure to pyrethroid pesticides, such as deltamethrin, cis-permethrin, and cypermethrin, can activate PXR [66], thereby influencing the metabolism of both exogenous compounds and endogenous steroid hormones [67]. Dysregulation of steroid hormone metabolism—including glucocorticoids, estrogens, and progestogens—is a well-recognized contributor to adverse pregnancy outcomes. Therefore, BF may disrupt endocrine homeostasis and impair reproductive function by binding to and activating PXR. In addition, BF also showed strong binding affinity to TRs. It has been demonstrated that exposure to BF interferes with TR signaling pathways in fish [68], and thyroid hormones are considered critical regulators of embryo implantation and early developmental processes [69]. These findings, together with our results, suggest that BF may affect pregnancy outcomes by disturbing maternal thyroid hormone homeostasis. ESRs are nuclear receptors, whereas GPER is membrane-bound. Both can be activated by E2. ESRs primarily mediate genomic effects, while GPER is involved in non-genomic signaling pathways, such as MAPK and PI3K cascades [70,71]. Our molecular docking results showed that BF exhibited comparable binding affinities to both ESRs and GPER, relative to E2. However, whether BF functions as an agonist or antagonist upon binding to these receptors remains unclear. Some studies have indicated that BF and permethrin exhibit estrogenic activity in vivo in fish, but display anti-estrogenic effects in vitro in human ovarian carcinoma cell lines (BG-1) [72]. Notably, ESRα may not be the primary target responsible for the anti-estrogenic effects of BF [73]. Therefore, whether BF elicits specific biological effects upon binding to ESRs or GPER in human uterine or placental tissues remains to be further investigated. In summary, dysfunction of ESRs and GPER may lead to disruption of downstream genomic and non-genomic signaling pathways [71], resulting in endocrine imbalance, uterine vascular dysfunction, and immune dysregulation [70,74,75]. In addition, other receptors such as AhR, PR, and ERRs also play essential roles in regulating female fertility by affecting embryonic development, endometrial decidualization, and trophoblast function [44,76]. Therefore, BF may contribute to adverse pregnancy outcomes by interfering with the function of these hormone receptors. Taken together, our findings suggest that BF can bind strongly to four hub proteins as well as multiple hormone receptors. However, the precise biological consequences of these interactions remain unclear and warrant further investigation through in vitro functional assays.

Despite uncovering potential regulatory mechanisms of hub genes in RIF and RPL and exploring the role of BF as an EDC, this study has several important limitations. First, the sample size was relatively small, which may limit the statistical power and generalizability of the results. Secondly, the conclusions of this study are primarily based on bioinformatics analyses, and the molecular docking results rely solely on computational predictions. In the absence of in vitro or in vivo experimental validation, the true biological relevance of the interactions between BF and its target proteins or receptors remains uncertain. Third, no exposure concentration data were included, making it difficult to assess the real-world physiological or environmental relevance of BF’s effects on the endometrium and immune cells. Further studies incorporating quantitative exposure assessments, functional experiments, and clinical hormone/immune data are needed to confirm and expand upon our findings.

## 5. Conclusions

Through bioinformatics analysis, this study identified *PTGS2*, *HMOX1*, *BCL2*, and *CYCS* as potential hub genes mediating BF-induced immunotoxic responses, which may contribute to adverse pregnancy outcomes. Furthermore, BF’s interaction with hormone receptors including PXR, ER, TR, PR, ERR, and GR may constitute a key mechanism contributing to its reproductive toxicity via endocrine disruption. These novel findings provide new theoretical insights into the reproductive toxicity of BF. However, further toxicological and mechanistic studies are needed to validate these mechanisms in detail.

## Figures and Tables

**Table 1:** Detailed dataset information.

GEO Number	Platform	Cohort	Type	Samples
GSE111974	GPL17077	RIF	RNA-seq	24 RIF and 24 controls
GSE165004	GPL16699	RPL	RNA-seq	24 RPL and 24 controls
GSE250130	GPL24676	RIF	scRNA-seq	10 RIF and 6 controls
GSE214607	GPL24676	RPL	scRNA-seq	3 RPL and 5 controls

**Table 2:** Molecular docking binding energy.

Receptor	Natural Ligand	Binding Energy (kcal/mol) (with Natural Ligand)	Binding Energy (kcal/mol) (with Bifenthrin)
PTGS2	-	-	−8.7
HMOX1	-	-	−8.3
CYCS	-	-	−6.9
BCL2	-	-	−7.6
ESRα	E2	−9.9	−8.1
ESRβ	E2	−11	−6.8
GPER	E2	−7.9	−7.6
PGR	Progesterone	−10.7	−6.6
GR	Cortisol	−8.1	−7.3
AhR	FICZ	−8.5	−6.5
TRα	T3	−9.3	−8.8
TRβ	T3	−7.8	−10.3
ERRα	-	-	−7.1
ERRβ	-	-	−6.6
ERRγ	-	-	−7.8
PXR	-	-	−10.6

Abbreviations: ESRα/ESRβ, Estrogen Receptor Alpha/Beta; GPER, G Protein-Coupled Estrogen Receptor; PGR, Progesterone Receptor; GR, Glucocorticoid Receptor; AhR, Aryl Hydrocarbon Receptor; TRα/TRβ, Thyroid Hormone Receptor Alpha/Beta; ERRα/ERRβ/ERRγ, Estrogen-Related Receptor Alpha/Beta/Gamma; PXR, Pregnane X Receptor; E2, 17β-Estradiol; FICZ, 6-Formylindolo [3,2-b] carbazole; T3, Triiodothyronine.

## Data Availability

The data used in this study are included in the article and its Supplementary Materials.

## Supplementary Materials

The following supporting information can be downloaded at: https://www.mdpi.com/article/10.3390/toxics13060454/s1. Figure S1: Batch correction in single-cell RNA sequencing analysis; Figure S2: Bar plots of cell type proportions based on single-cell RNA sequencing data; Figure S3: 3D and 2D visualizations of molecular docking between small molecules and target proteins; Table S1: 263 bifenthrin-related genes.
