# Supplementary material for: Exploring the Role of Bifenthrin in Recurrent Implantation Failure and Pregnancy Loss Through Network Toxicology and Molecular Docking
Source: Toxics. 2025 May 29;13(6):454. doi: 10.3390/toxics13060454 (PMC12196838; doi:10.3390/toxics13060454)
Supplement: Supplementary file 1 [file toxics-13-00454-s001.zip › toxics-3607678-supplementary.pdf]

Supplementary Figure S1.

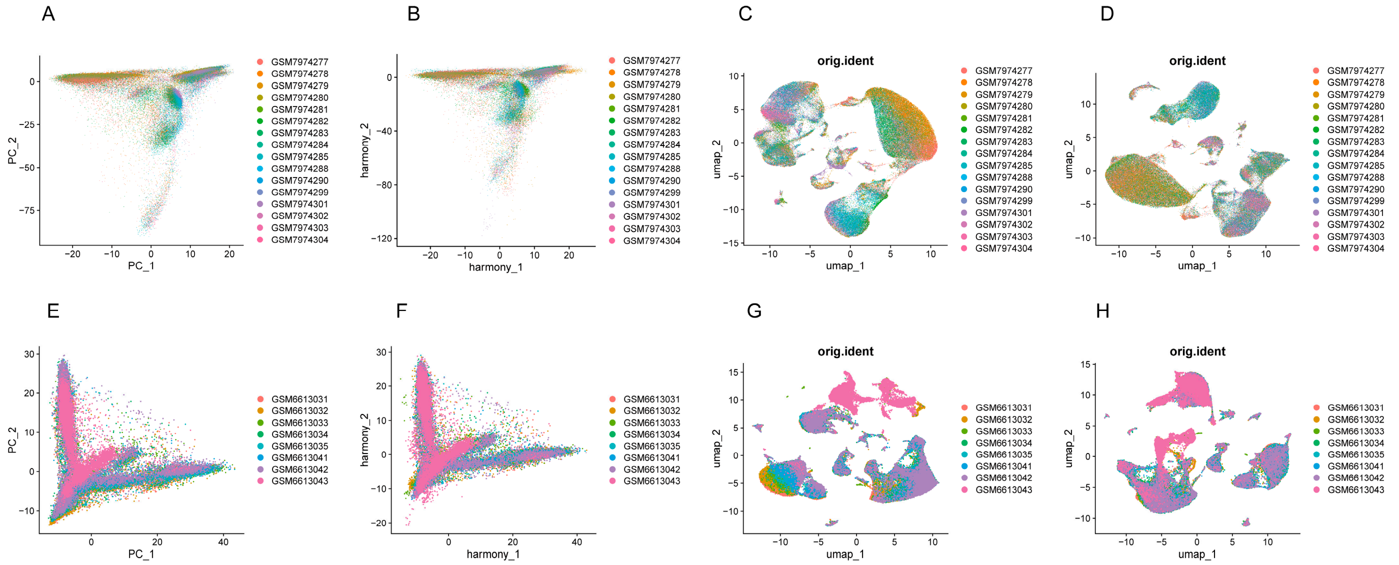

Supplementary Figure S1. Batch correction in single-cell RNA sequencing analysis. (A–B) Principal component (PC) plots of the RIF cohort before and after Harmony integration. (C–D) Clustering results of the RIF cohort before and after Harmony integration. (E–F) PC plots of the RPL cohort before and after Harmony integration. (G–H) Clustering results of the RPL cohort before and after Harmony integration.

Supplementary Figure S2.

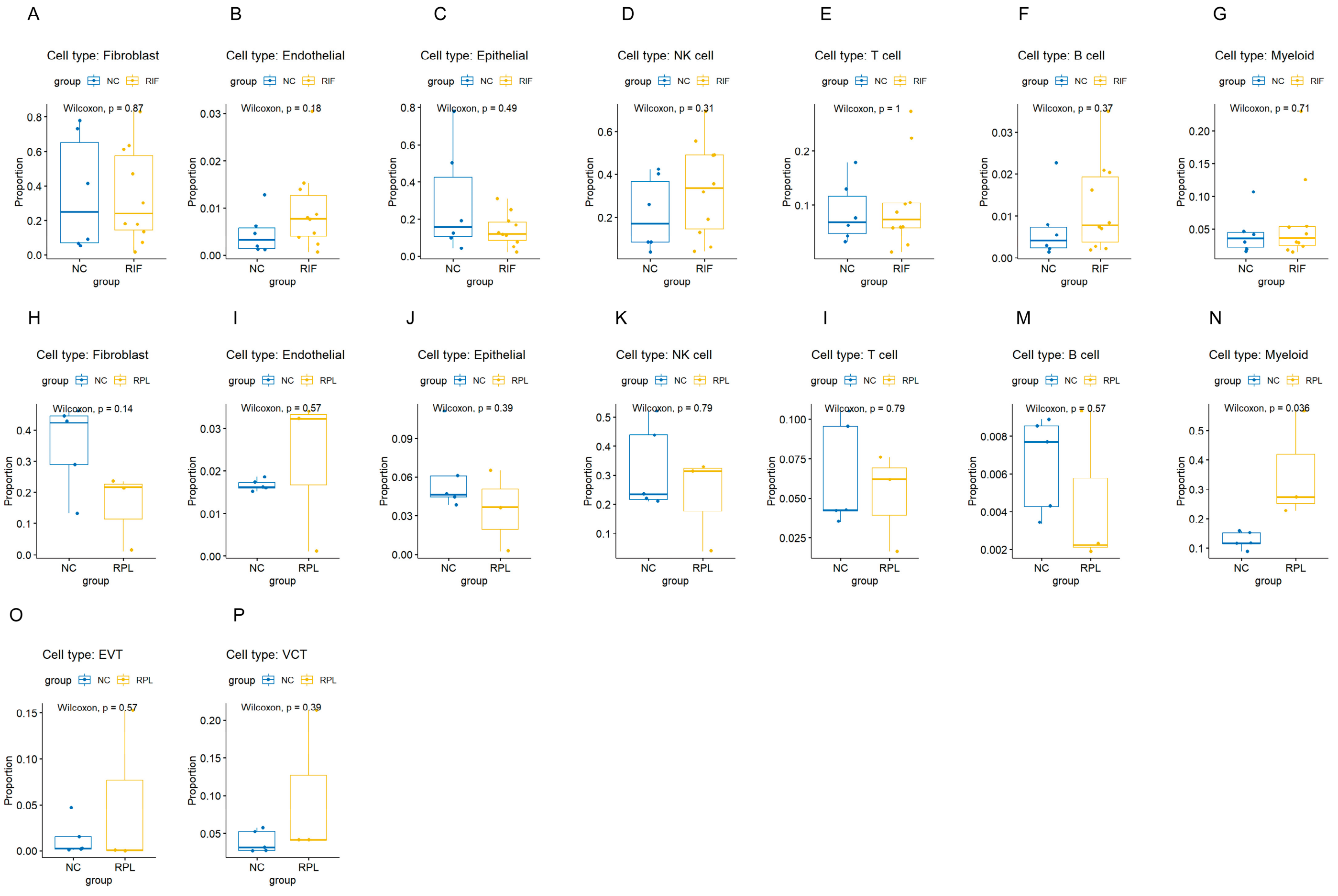

Supplementary Figure S2. Bar plots of cell type proportions based on single-cell RNA sequencing data. (A–G) RIF cohort; (H–P) RPL cohort. Statistical analysis was performed using the Wilcoxon test.

Supplementary Figure S3.

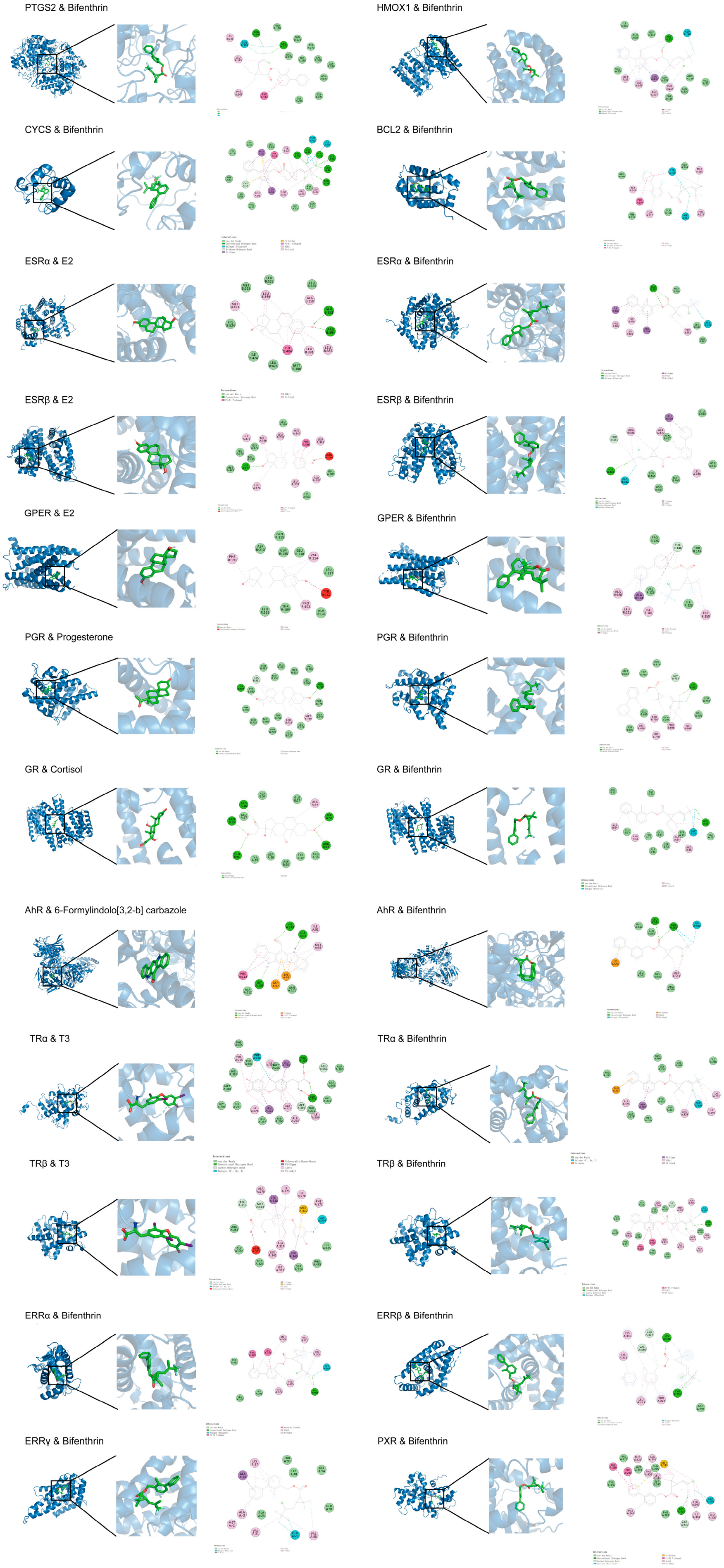

Supplementary Figure S3. 3D and 2D visualizations of molecular docking between small molecules and target proteins.

Supplementary Table S1. 263 BF-related targets derived from five databases.

263 Bifenthrin-related Genes							
ABCB1B	ACHE	ADH1	ADH5	AHR	AKT1	ALDH1A1	ALOX12
ALOX15	ALPL	APAF1	APOE	AREG	AR	ARNT	ASPA
ATP2A2A	BAIAP2L2	BAX	BCHE	BCL2	BID	BRCA1	CACNA1C
CACNA1SA	CACNA1SB	CASP3	CASP3A	CASP8	CASP9	CASPB	CAT
CCL14	CCL4	CCND1	CCNE1	CD36	CDK2	CDK6	CDKN1A
CEBPB	CES2	CES2A	CES2E	CGA	CHAT	CHRM1	CHRNA2
CPT1A	CRHB	CX3CL1	CXCL1	CXCL18B	CXCL8A	CYB5R3	CYCS
CYP11A1	CYP11B1	CYP11B2	CYP17A1	CYP19A1	CYP19A1A	CYP1A1	CYP21A2
CYP2C29	CYP2E1	CYP3A4	CYP4B1	DBI	DDIT3	DIO1	DIO2
EIF2S1	EIF4EBP1	EPHX1	ERBB2	EREG	ESR1	ESR2	ESR2A
ESR2B	ESR2.L	FAIM	FASL	FASN	FBP1	FGFR2	FLT1
FLT4	GGT1	GNRH1	GNRHR	GPER1	GPI1	GPX1	GPX2
GPX3	GSTA1	GSTA3	GSTD1	GSTM1	GSTM2	GSTM5	GSTP1
HK2	HLAG	HMOX1	HSD17B1	HSD17B3	HSD3B1	HSD3B2	HSPA1A
HSPA5	HSPA8	HSPB2	IL1B	IL2	IL4	IL6	KDR
LDLR	LEPR	MAP1LC3B	MAPK1	MAPK14	MAPK3	MAPK8	MGST2
MGST3	MIR122	MIR17	MIR34B	MIR34C	MIR449A	MIR449C	MT2
MT3	MTHFR	MTOR	NAT1	NFE2L2	NQO1	NR1H4	NR1I2
NR3C1	NR3C2	NR4A2	PCK1	PCNA	PGR	PKLR	PKM
PON1	PON3	PPARD	PPARG	PTGES	PTGS2	PTGS2B	PVALB
RARB	RELA	RHEB	RICTORA	RORA	RORC	RPS6	RPS6KB1
RPTOR	RUNX1	RYR1B	RYR2A	RYR3	SCARB1	SCD	SCN10A
SHBG	SOD1	SOD2	SQSTM1	SRC	SRD5A2	STAR	SULT1E1
TFF1	TGFB1	THRA	THRAA	THRB	TNF	TNFA	TP53
TRH	TRP53	TSHBA	TSHB	TSPO	VEGFAA	HSP90AA1	SCN1A
HSP90B1	BDNF	S1PR4	PTGER2	HTR1E	PLA2G1B	SLC6A4	APOBEC3A
ALOX5	CYP1A2	CTDSP1	NR2F2	CACNA1B	SLC6A3	TLR9	GPR35
TGM2	MIF	PTGS1	CHRM4	AKR1B1	CNR2	ABCB1	S1PR2
GALR3	CNR1	RIPK2	S1PR1	DRD5	CTSG	CYP2C9	SLC6A2
TAAR1	PTPN1	CDC25B	ELANE	NR1H3	ICMT	CHRM5	PTPN2
SCN5A	APOBEC3G	S1PR3	CHRM3	FDFT1	CES1	DNMT1	CA6
STS	CA5A	MAOA	S1PR5	KCNA3	PLIN5	CYP2C19	PDE4B
BCL2A1	CYP2D6	PTPN7	PLIN1	FFAR1	HDAC2	GRIN2B	
